# Sex differences in the association between episodic memory residual reserve index and change in executive function

**DOI:** 10.1016/j.nbas.2025.100146

**Published:** 2025-07-31

**Authors:** Cheyenne Chooi, Brandon E. Gavett, David Ames, Paul Maruff, Vincent Doré, Victor L. Villemagne, Pierrick Bourgeat, Ying Xia, Colin L. Masters, Ralph N. Martins, Kevin Taddei, Christopher C. Rowe, Michael Weinborn, Stephanie R. Rainey-Smith

**Affiliations:** a School of Psychological Science, University of Western Australia, 35 Stirling Highway, Crawley, Western Australia, Australia; b Department of Psychology, University of California, Davis, CA, USA; c National Ageing Research Institute, Royal Melbourne Hospital, Melbourne, Australia; d University of Melbourne Academic Unit for Psychiatry of Old Age, St George’s Hospital, Kew, Victoria, Australia; e Cogstate Ltd, Melbourne, Victoria, Australia; f CSIRO Health and Biosecurity/Australian e-Health Research Centre, Herston, Australia; g Department of Psychiatry, University of Pittsburgh, Pittsburgh, PA, USA; h The Florey Institute of Neuroscience and Mental Health, University of Melbourne, Melbourne, Victoria, Australia; i Department of Biomedical Sciences, Macquarie University, New South Wales, Australia; j School of Medical and Health Sciences, Edith Cowan University, Joondalup, Western Australia, Australia; k Department of Molecular Imaging and Therapy, Centre for PET, Austin Health, Heidelberg, Victoria, Australia; l Centre for Healthy Ageing, Health Futures Institute, Murdoch University, 90 South Street, Murdoch, Western Australia, Australia; m Australian Alzheimer’s Research Foundation, Sarich Neuroscience Research Institute, 8 Verdun Street, Nedlands, Western Australia, Australia; n Lifestyle Approaches Towards Cognitive Health Research Group, Murdoch University, 90 South Street, Murdoch, Western Australia, Australia

**Keywords:** Cognitive reserve, Residual reserve index, Sex differences, Alzheimer’s disease, Episodic memory, Beta-amyloid

## Abstract

Sex differences in cognitive reserve might contribute to females being disproportionately affected by Alzheimer’s disease (AD). We investigated sex differences in the protective effects of cognitive reserve, and whether brain beta-amyloid accounts for differences. Older adults (n = 997 from the Australian Imaging, Biomarkers and Lifestyle Study of Ageing) diagnosed as Cognitively Normal, Mild Cognitive Impairment, or AD at baseline were assessed every 18 months for up to a maximum of seven visits. Cognitive reserve was calculated from the variance in episodic memory not explained by demographic or brain measures. Executive functioning (EF) intercept and slope were regressed onto the main and interaction effects of cognitive reserve x brain integrity x sex, plus covariates (age, number of *APOE* ε4 alleles). A three-way interaction was observed between cognitive reserve, brain integrity, and sex on the EF slope. Females benefitted more than males from the protective effects of cognitive reserve at low levels of brain integrity. Sex differences in the protective effect of cognitive reserve were not moderated by brain beta-amyloid burden.

## 1 Introduction

Cognitive reserve is conceptualised as the adaptive ability of cognitive processes to sustain themselves in the event of brain aging, pathology, or injury [1]. Higher levels of cognitive reserve are believed to delay the onset of disease-associated clinical manifestations, such as Alzheimer’s disease (AD) dementia. Previous studies have observed higher baseline cognitive reserve predicts slower decline in executive function [2], an effect that was later found to be stronger in individuals with cerebrospinal fluid biomarkers of AD pathology [3]. Sex differences have been identified in AD, such that there appear to be different thresholds for clinical symptoms between males and females despite equivalent neuropathology [4]. Moreover, brain beta-amyloid (Aβ) plaque accumulation has been associated with the earlier development of brain tau deposition in females compared to males (e. g., [5], [6]). Although AD is more prevalent in females than males [7], little is known about whether the protective effect of cognitive reserve differs by sex. The present study uses a residual approach that defines cognitive reserve as the difference between observed versus predicted episodic memory called the residual reserve index [2]. Additionally, we sought to explore whether sex differences in residual reserve – if observed – are attributable to Aβ accumulation in the brain. These aims were investigated in a diagnostically heterogeneous sample of older adults from the Australian Imaging, Biomarkers, and Lifestyle (AIBL) flagship Study of Ageing.

### 1.1 Alzheimer’s Disease: Dementia and neuropathology

AD is an insidious process of abnormal protein aggregation and brain cell death; the dementia associated with AD accounts for approximately 70% of dementia cases [8]. Mild cognitive impairment (MCI) is defined as a state intermediate between normal cognition and AD [9]. Brain Aβ plaques are associated with increased risk of neurodegeneration and may catalyse the progression of other neurodegenerative processes [10].

#### 1.1.2 Sex differences in Alzheimer’s disease

To clarify terminology used in the current study, ‘sex’ differences typically refer to differences in biological characteristics stemming from underlying genetic and biological factors (e.g., sex chromosomes and sex hormones). When discussing sex differences, individuals are typically classified as ‘females’ or ‘males’ based on these biological characteristics. On the other hand, ‘gender’ differences typically refer to differences in the sociocultural expectations, constraints, roles, or norms that surround each gender; studies incorporating gender differences typically refer to individuals as women, men, intersex, or non-binary. The present study refers to self-reported sex differences, however we note that sex is not strictly binary nor mutually exclusive from gender (e.g., [[11], [12]]).

Previous studies report females are more susceptible to the harmful effects of AD neuropathology than males [[5], [13], [14], [15]]. Females account for two-thirds of AD diagnoses[16] and tend to have a faster transition from typical cognition and MCI to AD dementia [[17], [18]]. All else equal, brain tau aggregates tend to accumulate more rapidly in amyloid-positive females than amyloid-positive males [19]. Further, females with the APOE ɛ4 allele or higher levels of brain Aβ pathology show more rapid tau accumulation, brain atrophy, and cognitive decline over time relative to males[6]. Sex differences in residual reserve indices between males and females may indicate underlying sex-related biological mechanisms of the residual approach, e.g., brain Aβ accumulation, differences in sex hormones, or some other cause(s).

### 1.2 Cognitive reserve

Cognitive reserve has been associated with less rapid conversion from MCI to AD dementia [20]. To date, mechanisms of cognitive reserve in the context of AD are unclear. Previous studies have sought to clarify these mechanisms by exploring individual differences in ageing outcomes. The present study seeks to investigate the residual approach of measuring cognitive reserve and explore how it may differ, if at all, by sex and brain beta-amyloid status. The present study specifically investigates individual differences according to sex, as there is evidence of heterogenous outcomes in AD progression between older females and males [21]. Sex differences in the residual approach could explain why females are disproportionately affected by AD.

Previous measures of cognitive reserve vary between studies; many studies have attempted to capture an individual’s cognitive reserve using information such as premorbid intelligence, education, or occupational status/complexity. While such measures are often associated with more favourable outcomes in later life, these results are predominantly limited to cross-sectional analyses [22]. These cognitive reserve proxies are also static (i.e., measured at a single time point) and may not accurately reflect individual variations in abilities across time or the dynamic nature of cognitive reserve [[23], [24]].

#### 1.2.1 The residual reserve index

The residual approach [2] operationalises cognitive reserve as a residual of episodic memory through latent variable modelling. In accordance with previous studies, the present study will decompose the variance in episodic memory performance into orthogonal brain integrity and demographic factors; variance not explained by these factors will be our marker of cognitive reserve (i.e., residual reserve index). According to the residual approach, higher observed episodic memory than predicted by brain integrity and demographic factors (i.e., a positive residual reserve index) is indicative of higher cognitive reserve. Lower residual reserve index (i.e., a negative residual reserve index) would indicate lower observed episodic memory scores than predicted, and thus lower cognitive reserve levels [2].

### 1.3 Rationale and study aims

Few studies have investigated sex differences in cognitive reserve, and among these, there has been inconsistent operationalisation of cognitive reserve. To date, only one study has explored sex differences using a variation of the Reed et al. [2] residual approach [25]. Digma and colleagues [25] examined an amyloid-positive sample drawn from the Alzheimer's Disease Neuroimaging Initiative (ADNI) study and found that females had higher levels of cognitive reserve than males, thus providing preliminary evidence of sex differences in cognitive reserve measured using a variation of the residual method.

The current study extends previous research on the well-established interaction between the residual reserve index and brain integrity by factoring sex into the model. In accordance with these previous studies, episodic memory performance variance will be decomposed into orthogonal brain integrity and demographic factors; variance not explained by these factors will be our marker of cognitive reserve (i.e., residual reserve index). Our study aims to explore sex differences, and the potential underlying impact of brain Aβ burden, on the effect of residual reserve index against brain integrity using structural equation modelling analyses to explore changes in executive functioning over up to seven 18-month follow-up visits.

## 2 Methods

### 2.1 Participants

Participant data for this study were collected by the Australian Imaging, Biomarkers and Lifestyle (AIBL) flagship Study of Ageing. AIBL is an ongoing longitudinal study of AD and ageing that commenced in November 2006, with 18-monthly follow-up assessments post-baseline. AIBL participants are at least 60 years of age at study entry and are assigned to one of three clinical diagnosis groups following neuropsychological assessment: AD, MCI, or cognitively normal (CN). Individuals initially classified as CN who reported a history of medication use or other substances that could affect cognition had their diagnostic status further considered [26].

The current study used archival data from 997 AIBL participants (*M*_age_ = 72.79; *SD* = 6.59) who underwent at least one magnetic resonance imaging (MRI) brain scan and more than one neuropsychological assessment. See Fowler et al. [26] for greater detail regarding AIBL Study participant recruitment, inclusion and exclusion criteria, and assessments. Briefly, exclusion criteria included having: a history of schizophrenia or bipolar disorder, significant current depression, Parkinson’s disease, cancer (other than basal cell skin carcinoma) within the last two years, symptomatic stroke, uncontrolled diabetes, excessive regular alcohol use, or history of head injury with over one hour of post-traumatic amnesia.

The AIBL Study has been approved by the ethics committees of Austin Health, St Vincent’s Health, Ramsay Health Care and Edith Cowan University, and written informed consent was obtained from all volunteers prior to participation.

#### 2.1.1 Clinical diagnosis

The Clinical Dementia Rating (CDR; Morris, 1993) and the Mini-Mental State Examination (MMSE) [27] were used to assist with classification of individuals into one of three groups: CN, MCI, or AD. CN participants (MMSE score ≥ 26; CDR = 0) had no evidence of MCI, dementia, or clinically significant depression as measured using the Geriatric Depression Scale (GDS; score > 5/15; Sheikh and Yesavage [28]). A multidisciplinary team of psychiatrists, geriatricians, neuropsychologists, and neurologists conducted monthly clinical review panel meetings to discuss the diagnostic classification for all potential AD or MCI participants [29]. This included individuals with a probable baseline diagnosis of AD or MCI, and those initially classed as CN whose diagnostic status required further consideration post-baseline. MCI diagnoses were made according to a protocol based on the criteria of Winblad et al.[30], which are informed by the criteria of Petersen et al. [31]. Probable and possible AD (MMSE = 14 to 26; CDR = 0.5 or 1) was diagnosed using NINCDS-ADRDA and DSM-IV [32] criteria, with the panel blind to positron emission tomography (PET) neuroimaging results.

### 2.2 Cognitive measures

#### 2.2.2 Episodic memory (AIBL-Mem)

A composite episodic memory measure was derived from the tests of episodic memory administered as part of the AIBL Study. This composite measure was created using confirmatory factor analysis-based statistical harmonization. This AIBL memory (AIBL-Mem) composite score was psychometrically equated with ADNI-Mem [33], an episodic memory composite score derived from the Alzheimer's Disease Neuroimaging Initiative (ADNI). The memory variables from AIBL that were used to generate this composite score were three memory items from the MMSE, Logical Memory I and II (Story A) from the Wechsler Memory Scale – Revised [34], Rey Complex Figure Test Delay (RCFT) [35], and California Verbal Learning Task-II (CVLT-II) [36]. ADNI and AIBL memory composite scores could be harmonised as they had the MMSE memory items and Logical Memory II in common with one another. Higher AIBL-Mem scores indicated better episodic memory performance. The benefit of a harmonisation approach is improved comparability of variables with other studies, e.g., improved comparability with the findings of prior work by McKenzie et al. [3] who investigated the residual reserve index with ADNI data.

#### 2.2.3 Executive function (AIBL-EF)

A composite executive function (EF) measure was derived from the tests of executive functioning administered as part of the AIBL Study at all visits. Like the AIBL-Mem variable, the AIBL-EF composite measure was created using confirmatory factor analysis-based statistical harmonization to correspond to the ADNI-EF composite score [37]. The executive functioning measures from AIBL that were used to generate this composite score were category fluency (animals), letter fluency (FAS), fluency switching, WAIS-III Digit Symbol Coding [38], WAIS-III Digit Span Backward [38], and the Clock Drawing test. AIBL and ADNI had category fluency (animals), Digit Span Backward, Digit Symbol Coding, and Clock Drawing in common.

### 2.3 Demographic measures

Demographic information was collected during the screening visit that determines eligibility for participation in the AIBL Study. Individuals completed a self-report questionnaire about details such as years of education, age, and sex. Sex data were obtained from a single item where participants could report their sex from the following options: *female, male*.

### 2.4 Neuroimaging measures

#### 2.4.1 Magnetic resonance imaging

Brain integrity measures were obtained in AIBL using structural MRI. Consistent with previous studies using the residual reserve index (e.g., [3,2]) hippocampal, grey matter, and white matter hyperintensity (WMH) volumes were used as predictors of episodic memory in the decomposition of episodic memory (AIBL-Mem) variance.

Per published protocols [26], the MRI scans included a 3D MPRAGE (Magnetization Prepared Rapid Acquisition Gradient Echo) image (voxel size 1.2 × 1 × 1 mm^3^, repetition time/ echo time = 2300/ 2.98, flip angle = 9◦). A 3D T2-weighted Fluid Attenuation Inversion Recovery (FLAIR) sequence was also included in the image acquisition protocol, which was acquired using two different sets of parameters: 1) in-plane resolution 0.98 × 0.98 mm, slice thickness 0.9 mm, repetition time/ echo time/ inversion time = 6000/ 420/ 2100, flip angle = 120◦, field-of-view 240 × 256, and 176 slices; 2) in-plane resolution 0.5 × 0.5 mm with in-plane interpolation (factor of 2) enabled, slice thickness 1.0 mm, repetition time/ echo time/ inversion time = 5000/ 355/ 1800, flip angle = 120◦, field-of-view 256 × 256, and 160 slices.

MPRAGE images were segmented into white matter, grey matter, and cerebrospinal fluid using an implementation of the expectation maximization algorithm [39]. Hippocampal extraction was performed using a multi atlas approach based on the Harmonized Hippocampus Protocol [40]. All measures were corrected for total intracranial volume. FLAIR images were used to quantify the volume of WMH. WMH were automatically segmented using the HyperIntensity Segmentation Tool based on an ensemble of pre-trained neural network classifiers [41,42]. Periventricular and deep WMH were classified using the distance-based criteria described in [43]. WMH volumes were quantified in the common Montreal Neurological Institute space and normalised by the total intracranial volumes (TIV), i.e., WMH/TIV (%).

#### 2.4.2 Cerebral Beta-Amyloid

The AIBL study uses PET imaging coupled with one of five Aβ-binding ligands to quantify Aβ plaque burden in the brain. Aβ ligands include: [C11]Pittsburgh Compound B, [F18]Flutemetamol, [F18]Florbetaben, [F18]Florbetapir, and [F18]NAV4694. Methodology for each tracer has been described in full previously [44]. Briefly, the PET scans require a slow intravenous bolus administration over 30–40 sec of 10 mCi of high-specific activity Aβ ligand. Participants then rest, for 30–90 min depending on the ligand injected, prior to undergoing PET scanning.

PET images were analysed using CapAIBL and brain Aβ burden was expressed in the Centiloid (CL) scale [44]. The CL scale provides a single continuous scale across the different Aβ imaging tracers, where a value of 0 represents the typical brain Aβ burden in young controls, and 100 the typical brain Aβ burden seen in mild AD patients [45]. The CL value at a participant’s first MRI scan was used as a measure of their brain Aβ burden.

### 2.5 Statistical analyses

The hypothesis of this study was tested using a three-way interaction (cognitive reserve x brain integrity x sex). Statistical analyses involved creating latent brain and demographic variables, decomposing episodic memory, growth curve modelling, and the final analytical model. Analyses were conducted using Mplus version 8.4 [46]), and R version 4.0.4 [47]. For all structural equation models, fit was assessed based on the following fit statistics using recommended cut offs [48]: chi-square test of model fit, comparative fit index (CFI) [49], Tucker-Lewis Index (TLI) [50], the root mean square error of approximation (RMSEA)[51], the standardised root mean square residual (SRMR) [52]. Lower values are favoured on the following fit statistics: Akaike Information Criterion (AIC) [53], Bayesian Information Criterion (BIC) [54], and adjusted BIC (aBIC) [55]. Missing data were handled using full information maximum likelihood.

#### 2.5.1 Episodic memory variance decomposition

Cognitive reserve was operationalised according to the method described by Reed et al. [2] with some adjustments. Episodic memory was regressed onto brain and demographic measures to obtain the residual variance (i.e., episodic memory performance not explained by brain or demographic factors).

The latent composite brain variable (MEMB) captures variance in episodic memory attributed to grey matter, white matter hyperintensity, and hippocampal volumes. The brain MRI variables used in the current study were pre-adjusted for intracranial volume; this differs from Reed et al. [2], who regressed global grey matter and hippocampus volumes on intracranial volume within their simultaneously estimated model.

The latent composite demographic variable (MEMD) captures variance in episodic memory attributed to demographic factors, such as education. Unlike Reed et al. [2], we did not residualise sex out of the residual reserve index because the goal of this study was to determine whether the residual reserve index differs by sex. Further, we did not residualise race and ethnicity variables out of the residual reserve index due to the lack of racial and ethnic diversity in the AIBL cohort. Additionally, we noted that some participants’ first MRI scans did not always co-occur with their first cognitive assessments (some MRI scans came later); thus, we included additional methodological variables under the ‘demographics’ umbrella. We included the number of previous cognitive assessments, the time interval containing those previous assessments, and the interaction between number of previous assessments and time interval to account for differential exposure to the assessment, which could artificially inflate episodic memory scores if not accounted for. Our preliminary analysis found that two attrition variables – withdrawn status and deceased status (0 = no, 1 = yes) – were significantly associated with episodic memory outcomes such that individuals who had been in the study for a longer time were more likely to begin with higher cognition and undergo less rapid cognitive decline. Thus, in the current study confounding variance of both withdrawn status and deceased status were also extracted from the residual reserve index.

**Fig. 1:**
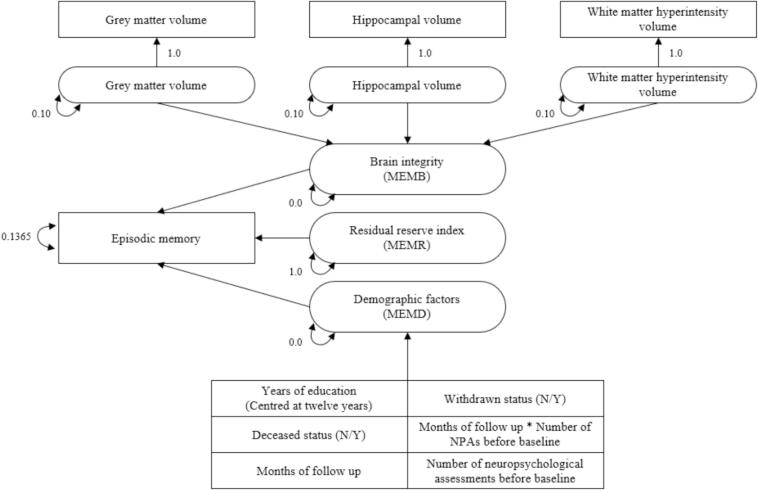
Decomposition of episodic memory to obtain three memory components: MEMB, MEMD, MEMR. Note. Squares indicate observed variables and ovals indicate latent variables. Brain factors, i.e., grey matter volume, hippocampal volume, and white matter hyperintensity volume, were used to freely estimate latent brain factors constrained to measurement error of 0.10. MEMB and MEMD are the linear combination of latent brain factors and observed demographic factors (i.e., education), respectively. The correlation between each of MEMB and MEMD, and MEMR were constrained to zero. Episodic memory error was set to 0.1365. Abbreviations: MEMB, brain integrity; MEMD, demographic factors; MEMR, residual reserve index; NPA, neuropsychological assessment.

After accounting for MEMB (the proportion of episodic memory variance attributable to brain variables) and MEMD (the proportion of episodic memory variance attributable to demographics), the remaining variance in episodic memory was adjusted for measurement error and defined as the residual reserve index (MEMR). Correlations between MEMB, MEMD, and MEMR were constrained to zero to ensure independence of episodic memory components. See Fig. 1 for a visual depiction of the residual model.

#### 2.5.3 Analytical model

**Fig. 2:**
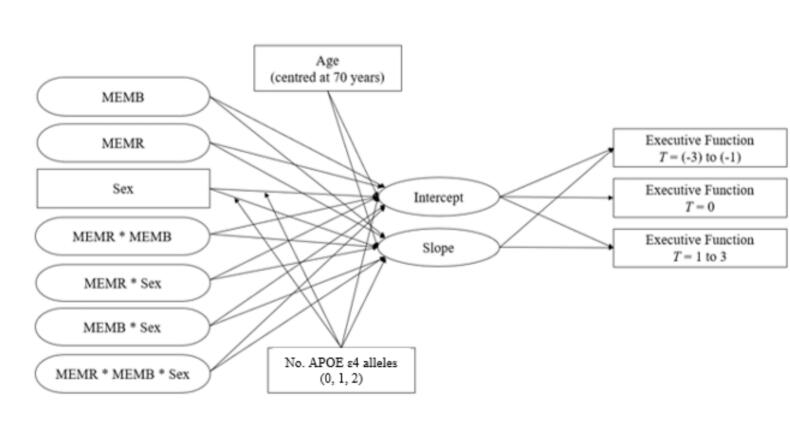
Analytical model regressing intercept and slope of executive function onto MEMR, MEMB, sex, and their interactions. Note. Rectangles indicate observed variables and ovals indicate latent variables. This figure visualises the analytical model used to investigate the study objective: does the protective effect of cognitive reserve against compromised brain integrity on executive functioning depend on sex. Brain integrity (MEMB; brain integrity), cognitive reserve (MEMR; the residual reserve index), sex, and the interaction between these three variables, were used as predictors of the intercept and slope of executive function. The executive functions scores at the first MRI were regressed onto the intercept and referred to as timepoint zero (T = 0). Longitudinal executive function were the executive functions scores from the visits three times points before (T = [-3] to [-1]) and after (T = 1 to 3) the first MRI visit. Age (centred at 70 years) and number of apolipoprotein E (APOE) ε4 carriers were also used as predictors. The sex * number of APOE ε4 alleles interaction effect was also controlled for. Not shown: the same model was repeated accounting for sex differences in cognitive reserve index that depend on brain beta-amyloid (Aβ) burden, represented by Centiloid value; this model incorporated a four-way interaction term examining residual reserve index x MEMB x sex x Aβ, as well as the main effect and interaction effects with Aβ.

Prior to hypothesis testing, AIBL-EF change over a period of up to 126 months was modelled with linear quadratic, and logarithmic growth functions to determine the most appropriate way to model change in the dependent variable. In the final analytical model, AIBL-EF intercept and slopes were regressed onto ‘baseline’ predictors to test our hypotheses. ‘Baseline’ cognitive measures for the current study corresponded to the neuropsychological assessment closest to a participant’s first MRI scan date (which did not necessarily correspond to the first assessment following study enrolment). Longitudinal executive functioning was inclusive of data ranging (at a maximum) from the three 18-month testing assessments before and after current study ‘baseline’, for a maximum total of up to seven visits per participant. Both the latent EF intercept (expected EF ability at the first MRI) and slope (rate of change in EF) were used as dependent variables within the same model. Intercept and slope were each regressed onto latent variables MEMB (brain integrity), MEMR (residual reserve index), sex, and all possible 2-way and 3-way interactions involving MEMD, MEMR, and sex (see Fig. 2). A significant 3-way interaction (MEMR x MEMB x sex) would be interpreted as support of the hypothesis that the protective effects of the residual reserve index differ by sex. If this interaction was found, it would be further probed with analysis of simple slopes to clarify the patterns contributing to this effect.

As mentioned in the introduction, age and the number of *APOE* ε4 alleles may influence cognitive outcomes; therefore, these factors were also included as predictors of the intercept and slope of EF. Given that the effect of *APOE* ε4 genotype on cognition may differ by sex (e. g., [6], the interaction between ε4 genotype (0 = zero ε4 alleles, 1 = heterozygotic ε4 alleles, 2 = homozygotic ε4 alleles) and sex was included in the model.

The same model was repeated incorporating brain Aβ main effects and interaction effects with other predictors of the initial model. In this second model, we were interested in the four-way interaction between the residual reserve index, MEMB, sex, and brain Aβ burden (measured on the Centiloid scale). A significant 4-way interaction would suggest that any sex differences in the protective effect of the residual reserve index were dependent upon the amount of Aβ burden present in the brain.

## 3 Results

Descriptive statistics are presented for the overall sample, and for the sample stratified by sex, in Table 1. Sex differences were identified in some of the variables. For example, females on average had higher baseline episodic memory and executive functioning scores, greater white matter hyperintensity volumes, and a greater proportion born in Australia, compared to males in the sample. Males on average had more years of education and more died during the study follow-up period.

**Table 1:** Participant Descriptive Statistics at First MRI Scan.

Variable	Overall	Male	Female	*p*	*SMD*
N	997	445	552		
Age (years) at first MRI; M (SD)	72.79 (6.60)	73.04 (6.52)	72.58 (6.66)	0.273	0.070
Education (years); M (SD)	12.95 (3.07)	13.33 (3.18)	12.63 (2.95)	0.002	0.228
APOE e4 carrier				0.972	0.015
0; n (%)	649 (65.1)	288 (64.7)	361 (65.4)		
1; n (%)	291 (29.2)	131 (29.4)	160 (29.0)		
2; n (%)	57 (5.7)	26 (5.8)	31 (5.6)		
Australian-born; n (%)	721 (74.9)	302 (70.9)	419 (78.2)	0.012	0.168
English primary language; n (%)	960 (99.4)	429 (99.8)	531 (99.1)	0.335	0.092
# study visits, total; M (SD)	4.80 (2.32)	4.76 (2.31)	4.83 (2.33)	0.616	0.032
# study visits @ first MRI; M (SD)	2.34 (1.62)	2.26 (1.54)	2.41 (1.68)	0.138	0.095
Months between first cognitive assessment and first MRI; M (SD)	24.37 (30.51)	22.44 (28.76)	25.94 (31.79)	0.072	0.115
Total number of MRI scans; M (SD)	2.16 (1.49)	2.25 (1.51)	2.09 (1.47)	0.090	0.108
Grey matter volume (mm^3^); M (SD)	454.17 (25.32)	455.20 (26.82)	453.34 (24.04)	0.248	0.073
Hippocampal volume (mm^3^); M (SD)	5.70 (0.72)	5.69 (0.76)	5.71 (0.69)	0.639	0.030
WMH volume (mm^3^); M (SD)	9.36 (13.41)	7.88 (10.40)	10.46 (15.19)	0.012	0.198
MMSE score; M (SD)	27.59 (3.39)	27.50 (3.34)	27.67 (3.43)	0.411	0.053
Withdrawn; n (%)	287 (28.8)	126 (28.3)	161 (29.2)	0.822	0.019
Deceased; n (%)	54 (5.4)	35 (7.9)	19 (3.4)	0.003	0.192
AIBL-Mem score; M (SD)	0.50 (0.91)	0.35 (0.85)	0.61 (0.94)	< 0.001	0.287
AIBL-EF score; M (SD)	0.82 (0.87)	0.73 (0.88)	0.89 (0.87)	0.005	0.179
PET brain amyloid, Centiloid; M (SD)	28.78 (42.83)	31.30 (43.34)	26.75 (42.35)	0.096	0.106
Diagnostic Group				0.134	0.128
CN; n (%)	758 (76.6)	326 (73.9)	432 (78.7)		
MCI; n (%)	136 (13.7)	71 (16.1)	65 (11.8)		
AD; n (%)	96 (9.7)	44 (10.0)	52 (9.5)		

*Note*. Significance tests correspond to sex differences in demographic factors. SMD = standardised mean difference. MRI = magnetic resonance imaging; WMH = white matter hyperintensity; MMSE = Mini-Mental State Examination; AIBL-Mem = episodic memory factor scores; AIBL-EF = executive functioning factor scores; PET = positron emission tomography; CN = Clinically Normal; MCI = Mild Cognitive Impairment; AD = Alzheimer’s Disease.

### 3.1 Growth modelling

A growth curve analysis was conducted to assess the best fit for the longitudinal EF data. Linear, quadratic, and logarithmic growth models fit well according to fit statistics (linear model: CFI = 0.993; TLI = 0.994, RMSEA = 0.036, 90 % CI [0.023, 0.049]; SRMR = 0.035, AIC = 5576.204, BIC = 5635.097, aBIC = 5596.984; quadratic model: CFI = 1.000; TLI = 1.000, RMSEA = 0.000, 90 % C.I. [0.000, 0.025]; SRMR = 0.017, AIC = 5553.546, BIC = 5632.070, aBIC = 5581.253; logarithmic: CFI = 0.961; TLI = 0.965, RMSEA = 0.071, 90 % C.I. [0.060, 0.083]; SRMR = 0.087, AIC = 5701.794, BIC = 5760.687, aBIC = 5722.575). All three models provided good fit to the data, and thus we chose to proceed with the linear model for parsimony, ease of interpretability, and comparability with similar studies[3,2].

### 3.2 Hypothesis testing

Sex differences in the protective effect of cognitive reserve were investigated using structural equation modelling with maximum likelihood estimation. The intercept and linear slope of EF were regressed onto the main and interaction effects of sex, MEMB, and MEMR, plus covariates. The regression coefficients are displayed in Table 2.

**Table 2:** Results of structural equation model predicting executive functioning intercept and slope.

	Intercept				Slope			
Variable	*est*	*SE*	*est/SE*	*p*	*est*	*SE*	*est/SE*	*p*
MEMR	0.413	0.032	13.082	0.000	0.028	0.010	2.716	0.007
MEMB	0.628	0.073	8.602	0.000	0.123	0.018	6.794	0.000
MEMD	0.268	0.044	6.024	0.000	0.025	0.007	3.680	0.000
Female sex	0.016	0.043	0.378	0.705	0.000	0.013	0.037	0.970
*APOE* ε4 carrier	0.040	0.042	0.940	0.347	−0.044	0.014	−3.169	0.002
Age at first MRI (centred at 70 years)	−0.008	0.003	−2.534	0.011	0.002	0.001	1.867	0.062
Female x *APOE* ε4 carrier	0.033	0.057	0.573	0.566	0.012	0.019	0.619	0.536
MEMR x MEMB	−0.110	0.039	−2.815	0.005	0.007	0.016	0.434	0.664
MEMR x Female sex	−0.047	0.041	−1.144	0.252	0.009	0.013	0.637	0.524
MEMB x Female sex	−0.039	0.059	−0.657	0.511	−0.004	0.019	−0.212	0.832
MEMR x MEMB x Female sex	−0.190	0.063	−3.009	0.003	−0.069	0.024	−2.827	0.005

*Note*. *est* = standardized parameter estimate; *SE* = standard error; *MEMR* = residual reserve index; *MEMB* = brain component of episodic memory variance; *MEMD* = demographic component of episodic memory variance. *APOE ε4* = apolipoprotein E ɛ4 allele; *MRI* = magnetic resonance imaging.

The intercept of EF was predicted by MEMR, MEMB, and age, such that having higher cognitive reserve, better structural brain health, and being of younger age at the first MRI was associated with higher EF intercept. The 3-way interaction between the residual reserve index, MEMB, and sex was significant, meaning that the degree to which the residual reserve index was protective against the effects of reduced brain integrity on the EF intercept differed depending on sex (*β* = -0.190, *p* = 0.003).

The slope of EF was predicted by MEMR, MEMB, age, number of *APOE* ε4 alleles , and the three-way interaction between the MEMR, MEMB, and sex. Being younger and being an *APOE* ε4 carrier were both predictive of more rapid decline in executive function when holding all other predictors constant. The 3-way interaction between MEMR, MEMB, and sex suggests that the degree to which cognitive reserve was protective against the effects of reduced brain integrity on EF slope differed depending on sex (*β* = -0.069, *p* = 0.005).

**Fig. 3:**
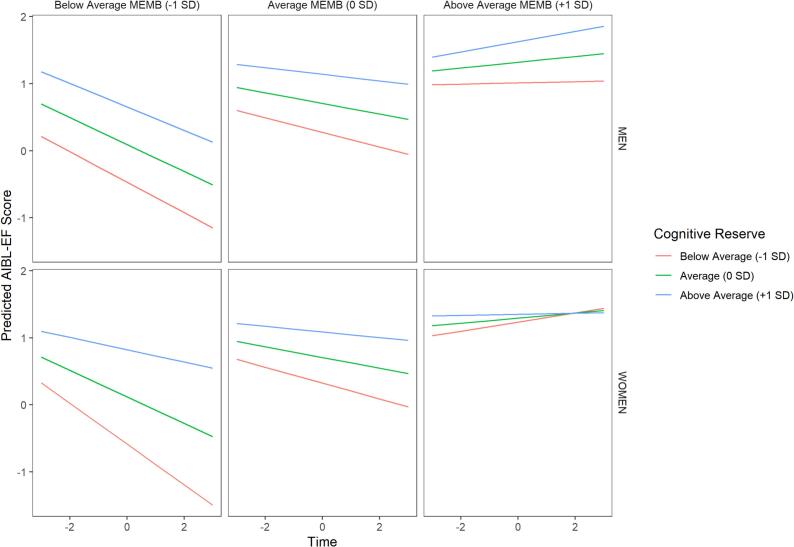
Model-predicted executive function performance as a function of MEMR, MEMB, and sex. *Note*. Figure shows models of AIBL-EF slopes split across different levels of cognitive reserve over time, determined using structural equation modelling, whereby Time 0 represents the AIBL-EF intercept, i.e., the AIBL-EF score at participants’ first MRI. Vertical facets correspond to below average (−1 standard deviation; SD), average (0 SD), and above average (+1 SD) levels of brain integrity (MEMB). Horizontal facets correspond to males (top row) and females (bottom row). Predicted executive functioning (AIBL-EF; i.e., ADNI-AIBL harmonised executive function scores) slopes over time are split across below average (−1 SD; red line), average (0 SD; green line), and above average (+1 SD; blue line) levels of cognitive reserve (i.e., residual reserve index; MEMR). (For interpretation of the references to colour in this figure legend, the reader is referred to the web version of this article.)

To further probe these significant interaction effects, model-predicted trajectories of EF scores were plotted as a function of MEMB, MEMR, and sex. For graphical purposes, MEMR and MEMB were categorized as low (1 SD below the mean), average (0 SD), and high (1 SD above the mean), as shown in Fig. 3. The data in Fig. 3 reflects how EF would be expected to change as a function of MEMB, MEMR, and sex for a reference participant: a 70-year-old *APOE* ε4-negative person with 12 years of education and average standing on the demographics composite variable (MEMD). Sex differences were apparent across different levels of cognitive reserve and brain volume. When brain volume was high (right facets), both males and females were predicted to demonstrate positive EF slopes (likely reflecting practice effects). However, males appeared to benefit more from higher cognitive reserve than females with above average brain volume. On the other hand, the protective effect of cognitive reserve against the negative effects of below average brain volume on EF slopes differed by sex, such that females received greater benefit from high levels of cognitive reserve when brain volume was low.

The structural equation modelling process was repeated to investigate whether there are underlying effects of brain Aβ burden on this three-way interaction, by including a four-way interaction incorporating the Centiloid measure. The regression coefficients are displayed in Table 3. In this second model, the four-way interaction between sex, MEMB, MEMR, and brain Aβ was of interest, and was found to lack statistical significance on both EF intercept (*β* = -0.001, *p* = 0.475) and slope (*β* = 0.000, *p* = 0.634). The results suggest the sex differences in the protective effect of cognitive reserve described above did not differ depending on underlying levels of brain Aβ.

**Table 3:** Centiloid brain beta-amyloid structural equation model results.

	Intercept				Slope			
Variable	*est*	*SE*	*est/SE*	*p*	*est*	*SE*	*est/SE*	*p*
MEMR	0.409	0.044	9.286	0.000	−0.005	0.014	−0.372	0.710
MEMB	0.568	0.079	7.171	0.000	0.066	0.020	3.307	0.001
MEMD	0.264	0.045	5.845	0.000	0.021	0.006	3.332	0.001
Female sex	−0.008	0.051	−0.154	0.878	−0.013	0.015	−0.869	0.385
A*β*	0.000	0.001	0.363	0.717	0.000	0.000	−1.415	0.157
*APOE* ε4 carrier	−0.117	0.066	−1.785	0.074	−0.030	0.021	−1.452	0.147
Age at first MRI (centred at 70 years)	−0.009	0.003	−3.028	0.002	0.002	0.001	1.744	0.081
MEMR x MEMB	−0.107	0.050	−2.120	0.034	0.047	0.018	2.633	0.008
MEMR x Female sex	−0.075	0.055	−1.371	0.170	0.035	0.017	2.019	0.043
MEMR x A*β*	0.001	0.001	1.491	0.136	0.000	0.000	1.405	0.160
MEMB x Female sex	0.052	0.076	0.676	0.499	0.037	0.025	1.486	0.137
MEMB x A*β*	0.003	0.001	3.034	0.002	0.001	0.000	2.841	0.005
Female sex x A*β*	−0.001	0.001	−0.546	0.585	0.000	0.000	0.113	0.910
Female sex x *APOE* ε4 carrier	0.121	0.085	1.421	0.155	0.019	0.027	0.700	0.484
A*β* x *APOE* ε4 carrier	0.004	0.001	3.638	0.000	0.000	0.000	0.218	0.827
MEMR x MEMB x Female sex	−0.142	0.083	−1.722	0.085	−0.105	0.029	−3.610	0.000
MEMR x MEMB x A*β*	0.001	0.001	1.489	0.136	0.000	0.000	−0.104	0.917
MEMR x Female sex x A*β*	0.001	0.001	0.964	0.335	0.000	0.000	−0.452	0.651
MEMB x Female sex x A*β*	−0.003	0.001	−2.272	0.023	−0.001	0.001	−1.674	0.094
Female sex x A*β* x *APOE* ε4 carrier	−0.002	0.001	−1.141	0.254	0.000	0.000	−0.432	0.666
MEMR x MEMB x Female sex x A*β*	−0.001	0.001	−0.715	0.475	0.000	0.001	0.476	0.634

*Note*. *est* = standardized parameter estimate; *SE* = standard error; *MEMR* = residual reserve index; *MEMB* = brain component of episodic memory variance; *MEMD* = demographic component of episodic memory variance; *Aβ* = positron-emission-tomography-imaged brain beta-amyloid, Centiloid; *APOE ε4* = apolipoprotein E ɛ4; *MRI* = magnetic resonance imaging.

## 4 Discussion

The protective effects of cognitive reserve against late-life cognitive decline have been well-documented. However, it is unclear whether these protective effects manifest differently in females versus males. The results from the current study suggest that females in our sample had – on average – higher standing on the residual reserve index (i.e., our estimate of cognitive reserve) than males, and the protective effects of cognitive reserve also differed by sex. However, brain Aβ burden did not appear to be a contributing factor to these observed sex differences. Our finding that females on average had higher levels of cognitive reserve is consistent with a study by Digma and colleagues [25]. Digma et al. [25] suggest this may be attributed to females’ verbal episodic memory advantage (e.g., [56]). Females have higher levels of cognitive reserve – i.e., episodic memory that is not attributed to brain and demographic factors.

### 4.1 Sex differences findings and Interpretation

Our study extends previous work by finding that females with higher risk of AD experience greater benefit from the protective ability of cognitive reserve. On the other hand, males experience greater benefit from cognitive reserve’s protective ability when their brains are already healthy. Our results could indicate a sex-specific biological mechanism by which cognitive reserve confers protection in females versus males in the presence of neurodegeneration.

The stronger association between cognitive reserve and favourable executive function outcomes in females may reflect a necessary adaptation in response to female-specific biological changes. Most notably, there are significant sex differences in endocrinological processes throughout the life course. Males have relatively stable levels of testosterone over their post-pubertal lifetime with a progressive, gradual transition over several decades into hypogonadism [57,58]. In contrast, females who are post-menarche must adapt to a variety of hormonal fluctuations such as regular cyclical changes during phases of menses, pregnancy, and menopause (e.g.,[59]). In the context of older adults, post-menopausal females have ceased gonadal synthesis of oestradiol resulting in a depletion in protective circulating oestradiol levels, which has been proposed as a potential mechanism leading to increased prevalence of AD in females [60].

Previous studies also suggest older female brains may be more susceptible to the development of metabolic and neurodegenerative disorders. For example, protective responses by microglia in female brains may recruit more pro-inflammatory mechanisms (e.g., lipid metabolism) against neuropathology while microglia in male brains may adopt more anti-inflammatory mechanisms (e.g., protein metabolism; [61]. Although an effective defence against neuropathology, pro-inflammatory mechanisms may inadvertently lead to greater neurological damage to remaining healthy brain structures and functions; females have a higher prevalence of conditions (e.g., auto-immune disorders, cardiovascular disease, type 2 diabetes mellitus) that contribute to pro-inflammatory states [62]. Despite having on average harsher neurological environments than males, females are often reported to have comparable or advantage in measures of cognitive performance (e.g., [56]), suggesting a role for cognitive reserve and resilience.

### 4.2 Brain Beta-Amyloid plaques findings & Interpretation

Previous studies suggest that cognitive reserve confers protection predominantly in the event of clinically significant neuropathology[3]; therefore, we explored the role of brain Aβ burden in relation to sex differences in cognitive reserve. We hypothesised that the harsher neurodegenerative effect of brain Aβ burden in females could be driving an adaptive mechanism resulting in more protection from cognitive reserve, however, our study did not support a role of brain Aβ burden in cognitive reserve’s differential protective effects in females versus males.

This seems to contradict the literature, which suggests brain beta-amyloid effects are more detrimental in females compared to males when cognitive reserve was low [59]. The role of brain Aβ burden in the context of cognitive reserve could potentially be explained by the amyloid tau neurodegeneration (ATN) model [63]. The ATN model describes brain Aβ accumulation as a catalyst for subsequent processes that have a stronger relationship with cognitive decline, e.g., tau hyperphosphorylation leading to neurofibrillary tangle accumulation and subsequent neurodegeneration. Support for the ATN model was found by Digma et al. [25] which showed protection in the context of tau. Thus, brain Aβ burden may not meet the threshold to ‘activate’ cognitive reserve’s protective effect; cognitive reserve may instead ‘activate’ in response to later processes such as tau phosphorylation and neurodegeneration.

### 4.3 Limitations

As with all research, it is important that this study be interpreted in the context of several limitations. Firstly, the current study was conducted in the context of predominantly highly educated, Caucasian older adults, which limits the generalisability of our findings to populations of comparable demographics. Secondly, while executive function measures are useful in aiding the diagnosis of various forms of dementia, they are not the only indicators of AD risk; while individuals with dementia or brain lesions may perform poorly on tests of executive function, this is does not necessarily tell us about outcomes related to their activities of daily living, quality of life, mental health, and overall everyday functioning. Future studies could look at the association between the residual reserve index and other measures of everyday function to understand broader implications of the residual reserve index’s effect.

#### 4.3.1 Residual reserve index

We note the method of estimating the residual reserve index (e.g., [[64], [65], [66]]) would, at a statistical level, classify cognitive reserve as prediction error[[67], [68]]. We attempted to delineate biologically relevant variability from measurement error by following Reed et al.’s [2] approach of specifying a known episodic memory measurement error. While this approach is not without limitations, it means we can avoid contaminating the residual with variance attributable to measurement error. We also note that residual scores may also have issues of high collinearity with the cognitive measures from which it was obtained[69]; the present study mitigates this by using different cognitive domains for predictor (i.e., episodic memory) and outcome (i.e., executive function) variables. Additionally, sex differences in cognitive reserve may reflect the well-established verbal memory advantage in females.

#### 4.3.2 Decomposition of episodic memory

Further, we note the validity of the residual approach is largely dependent on the specific predictor and outcome measures used to create the residual reserve index [70]. The residual approach involves the decomposition of a cognitive measure into variance that can be attributed to brain factors, demographics factors, and unexplained factors. While there is no specific cognitive domain generally agreed upon for deriving the residual reserve index, the residual approach by Reed and colleagues [2] initially used episodic memory for its strong association with aging. Additionally, measures of episodic memory are sensitive to both typical and atypical changes in age (e.g., [[71], [72], [73], [74]]),. Several studies have empirically demonstrated the use of episodic memory in the residual approach (e.g., [[2], [3], [23], [24], [75]]).

#### 4.3.3 Sex versus gender differences

Although the residual reserve index is generally less biased by sociocultural factors than other cognitive reserve proxies (e.g., historically reduced access to education for women), there may be confounding variables related to mid- and early-life that we cannot account for in the current dataset. As mentioned previously, sex and gender differences are not mutually exclusive; although the present study seeks to investigate differences attributed to the biological sex in females versus males, there is likely an influence of gender-related differences(e.g., [11]). Sex differences observed in the current sample could be influenced by societal differences where defined gender roles and expectations were more pertinent. The results of the present study permit further investigations into both sex-related and gender-related mechanisms underlying observed differences between males and females.

### 4.4 Implications

Despite the described limitations, there are multiple aspects that give us confidence in our findings, including the use of a highly characterised cohort of older adults and rigorous statistical approach. Our research may have clinical implications for informing sex-specific cognitive reserve interventions. Our findings suggest that females would benefit more from interventions that focus on increasing their cognitive reserve by improving episodic memory functioning (e.g., cognitive interventions). On the other hand, males appear to benefit most from cognitive reserve at higher brain volumes; this could suggest that interventions for males would focus on early lifestyle modifications or biological interventions that maintain or support brain integrity (brain maintenance).

However, for existing and emerging older adult populations the critical period to effectively build cognitive reserve through cognitive or lifestyle interventions may have already elapsed, at which point, biological interventions that increase the protective ability of cognitive reserve would be beneficial across both sexes. Some avenues of research into sex differences could investigate how cognitive reserve differs depending on endocrinological, metabolic, or inflammatory factors, which show well-established sex differences in older age. Further research is needed to understand the sex-specific mechanisms by which cognitive reserve is more protective in females compared to males to inform the development of such treatment approaches.

### 4.5 Conclusion

Overall, the current study found sex differences in both the levels and protective ability of cognitive reserve against low brain integrity levels. This interaction was not moderated by brain Aβ burden levels, and therefore may be attributed to other sex-related biological processes. Understanding the biological mechanisms by which cognitive reserve elicits protective effects could inform future interventions to optimally maximise protective benefit from cognitive reserve in females and males.

## Funding Statement

The AIBL Study (www.AIBL.csiro.au) is a consortium between 10.13039/501100020211Austin Health, 10.13039/501100000943CSIRO, 10.13039/501100001798Edith Cowan University, the 10.13039/501100024218Florey Institute (10.13039/501100001782The University of Melbourne), and the National Ageing Research Institute. The study has received partial financial support from the Alzheimer’s Association (US), the Alzheimer’s Drug Discovery Foundation, an Anonymous foundation, the 10.13039/100008716Science and Industry Endowment Fund, the Dementia Collaborative Research Centres, the 10.13039/501100004752Victorian Government’s Operational Infrastructure Support program, the Australian Alzheimer’s Research Foundation, the 10.13039/501100000925National Health and Medical Research Council (NHMRC), and 10.13039/501100016005The Yulgilbar Foundation. Numerous commercial interactions have supported data collection and analyses. In-kind support has also been provided by Sir Charles 10.13039/100012267Gairdner Hospital, Cogstate Ltd, Hollywood Private Hospital, 10.13039/501100001782The University of Melbourne, and St Vincent’s Hospital. SRRS is supported by an 10.13039/501100000925NHMRC Investigator Grant GNT1197315).

## CRediT authorship contribution statement

**Cheyenne Chooi:** Writing – original draft, Visualization, Methodology, Investigation, Formal analysis. **Brandon E. Gavett:** Writing – review & editing, Visualization, Validation, Supervision, Software, Project administration, Methodology, Investigation, Formal analysis, Conceptualization. **David Ames:** Writing – review & editing, Resources. **Paul Maruff:** Writing – review & editing, Resources, Data curation, Conceptualization. **Vincent Doré:** Writing – review & editing, Resources, Data curation. **Victor L. Villemagne:** Writing – review & editing, Resources, Data curation. **Pierrick Bourgeat:** Writing – review & editing, Resources, Data curation. **Ying Xia:** Writing – review & editing, Resources, Data curation. **Colin L. Masters:** Writing – review & editing, Resources, Data curation. **Ralph N. Martins:** Writing – review & editing, Resources, Data curation. **Kevin Taddei:** Writing – review & editing, Resources, Data curation. **Christopher C. Rowe:** Writing – review & editing. **Michael Weinborn:** Writing – review & editing, Validation, Supervision, Resources, Project administration, Methodology, Conceptualization. **Stephanie R. Rainey-Smith:** Writing – review & editing, Validation, Supervision, Resources, Project administration, Methodology, Conceptualization.

## Declaration of competing interest

The authors declare the following financial interests/personal relationships which may be considered as potential competing interests: C.C., B.E.G., D.A., V.D., P.B., Y.X., K.T., M.W., and S.R.R.S. report no disclosures. P.M. is a full-time employee of Cogstate Ltd. V.L.V. is and has been a consultant or paid speaker at sponsored conference sessions for Eli Lilly, Life Molecular Imaging, ACE Barcelona, and IXICO. C.L.M. is an advisor to Prana Biotechnology Ltd and a consultant to Eli Lilly. R.N.M. is founder of, and owns stock in, Alzhyme, and is a co-founder of the KaRa Institute of Neurological Diseases. C.C.R. has served on scientific advisory boards for Bayer Pharma, Elan Corporation, GE Healthcare, and AstraZeneca, has received speaker honoraria from Bayer Pharma and GE Healthcare, and has received research support from Bayer Pharma, GE Healthcare, Piramal Lifesciences and Avid Radiopharmaceuticals.
